# Second-generation H1 antihistamines for the treatment of chronic urticaria: a network meta-analysis

**DOI:** 10.3389/fmed.2026.1880701

**Published:** 2026-08-13

**Authors:** Zixuan Li, Juan Zhang, Yaqi Zheng, Mingyan Zhang, Wenkang Du, Yajun Zhang, Xiaochao Gao, Qing Zhu, Jinxu Qi, Zuotao Zhao, Lijuan Liu

**Affiliations:** 1 Department of Dermatology, The First Hospital of Hebei Medical University, Shijiazhuang, Hebei, China; 2 Subcenter of National Clinical Research Center for Skin and Immune Diseases, Shijiazhuang, Hebei, China; 3 Hebei Provincial Innovation Center of Dermatology and Medical Cosmetology Technology, Shijiazhuang, Hebei, China; 4 Department of Epidemiology and Statistics, School of Public Health, Hebei Medical University, Hebei Province Key Laboratory of Environment and Human Health, Shijiazhuang, China; 5 Department of Dermatology and Venerology, Peking University First Hospital, Beijing, China; 6 Department of Dermatology, Tianjin Academy of Traditional Chinese Medicine Affiliated Hospital, Tianjin Institute of Integrative Dermatology, Tianjin, China

**Keywords:** antihistamines, chronic spontaneous urticaria, chronic urticaria, licensed dose, network meta-analysis, second-generation H1 antihistamines, treatment comparison

## Abstract

**Objective:**

To evaluate the efficacy and safety of 10 commonly used second-generation H1 antihistamines (cetirizine, levocetirizine, loratadine, desloratadine, ebastine, bilastine, olopatadine, rupatadine, fexofenadine, and mizolastine) at licensed doses in treating chronic urticaria using network meta-analysis.

**Methods:**

Seven electronic databases were searched, including PubMed, Embase, Web of Science, Cochrane Library, China National Knowledge Infrastructure (CNKI), Wanfang Data Knowledge Service Platform, and Chinese Biomedical Literature Service System. Relevant literature from inception to the end of December 2025 was retrieved. Studies meeting the criteria were selected, and network meta-analysis was performed using Stata 16 software.

**Results:**

A total of 54 studies involving 7,290 patients were included. Network meta-analysis indicated that olopatadine, mizolastine, bilastine, and levocetirizine were more effective than placebo in improving total symptom scores. All 10 drugs were superior to placebo in reducing itching scores, although only fexofenadine achieved statistical significance. For reducing wheal scores, ebastine was significantly more effective than mizolastine and loratadine. Regarding adverse reactions, no significant differences were observed between any of the drugs and placebo, with fexofenadine having the lowest adverse reaction rate. Sensitivity analyses excluding highrisk studies confirmed the robustness of findings across all outcomes.

**Conclusion:**

Based on current evidence, olopatadine at licensed dose exhibited the best efficacy in reducing total symptom scores. Fexofenadine was most effective for improving itching scores and ebastine was optimal for reducing wheal scores. In addition, fexofenadine had the lowest incidence of adverse reactions. However, the conclusions of this study still need to be validated by further high-quality randomized controlled trials.

## 1 Introduction

Chronic urticaria (CU) is a common dermatological disease, characterized by spontaneous wheals, angioedema or both. Its etiology is complex, prone to recurrence, and accompanied by intense itching, significantly impacting patients’ quality of life (1, 2). Studies have shown substantial regional variations in CU prevalence, the prevalence is approximately 0.5–1%, with an increasing incidence (3). The second-generation antihistamines (SgAHs) are the first-line treatment for CU, but many options exist with varying efficacy (1, 4). While some meta-analyses compare the efficacy and adverse effects of two sgAHs, comparisons involving multiple drugs are currently limited. Ten antihistamines (ebastine, loratadine, desloratadine, cetirizine, levocetirizine, mizolastine, fexofenadine, olopatadine, rupatadine, and bilastine) were selected for this study. The available randomized evidence was analyzed using network meta-analysis (NMA) to assess the efficacy and safety of these drugs. A ranking based on efficacy and safety was subsequently generated, providing evidence-based medical recommendations for rational and efficient drug use.

## 2 Materials and methods

### 2.1 Data sources and search strategy

We searched 7 electronic databases in English and Chinese, including PubMed, Embase, Web of Science, Cochrane Library, China National Knowledge Infrastructure (CNKI), Wanfang Data Knowledge Service Platform, and Chinese Biomedical Literature Service System. CNKI, Wanfang, and Chinese Biomedical Literature Service System are the largest academic databases in China, covering Chinese-language medical journals. The search included literature from the inception date to the end of December 2025. Only studies published in English or Chinese were included. No restrictions were placed on publication date or country of origin. This approach aligns with PRISMA guidelines and ensures comprehensive retrieval of the global evidence base, rather than focusing on a specific population.

### 2.2 Study selection and outcomes

Duplicate publications were removed. Two reviewers independently screened the titles and abstracts to exclude obviously irrelevant studies. The full texts of potentially eligible studies were then evaluated against the predefined inclusion and exclusion criteria to determine the final included studies. Randomized controlled trials (RCTs) meeting the following inclusion criteria were included: (1) Chronic urticaria (CU) patients aged over 12 years; (2) Intervention with second-generation antihistamines (sgAHs) (bilastine, cetirizine, desloratadine, ebastine, fexofenadine, levocetirizine, loratadine, mizolastine, olopatadine, placebo, rupatadine); (3) Comparison of sgAHs to placebo or comparisons between two sgAHs; (4) Outcome measures included total symptom score (TSS), itch score, wheal score, and adverse reaction rate. Studies with insufficiently described data or inaccessible full texts were excluded. All outcomes were measured at 2–4 weeks after randomization. If there were multiple visits within these intervals, the visit with the longest interval was selected. For example, if post-treatment visits occurred weekly, the outcome assessment would be conducted at week 4 rather than week 2 or 3. Any outcome measurements before or after this specified period were not considered.

### 2.3 Data extraction and risk of bias assessment

Two reviewers independently extracted data from the included studies using a predefined data extraction form, including the first author, publication year, study region, intervention, sample size, randomization method, blinding, and outcome measures (TSS, itch score, wheal score, and adverse reaction rate). The Cochrane risk-of-bias tool was used to assess the risk of bias in the included RCTs, focusing on random sequence generation, allocation concealment, blinding, incomplete outcome data, selective reporting, and other biases. Risk of bias was categorized as “low risk,” “high risk,” or “uncertain risk.” Disagreements were resolved by a third reviewer.

### 2.4 Data synthesis and analysis

This study conducted a network meta-analysis (NMA) within a frequentist framework to compare the efficacy and safety of sgAHs with placebo using Stata 16. All continuous variables were analyzed using the mean difference (MD) and its 95% confidence interval (CI). To account for heterogeneity in study scoring, standardized mean differences (SMDs) and their 95% CIs were used to calculate pooled effect sizes. For SMD, the line of no effect is 0; a positive SMD indicates superiority over placebo, and statistical significance is achieved when the 95% CI does not include 0. Binary outcome were summarized using odds ratios (ORs) and 95% CI. For OR, the line of no effect is 1. NMA integrates direct and indirect evidence from multiple RCTs within a single analysis. Direct evidence is obtained directly from existing research, while indirect evidence is derived through statistical algorithms. In addition to the transitivity assumption, the consistency between direct and indirect evidence must be evaluated. The presence of inconsistency is typically considered a statistical manifestation of intransitivity. We analyzed and compared consistency and inconsistency models. The node-splitting method is employed to assess inconsistency by locally comparing direct and indirect evidence, helping researchers identify problematic nodes and enhance the credibility of results. The efficacy of treatments was ranked using the surface under the cumulative ranking curve (SUCRA). SUCRA values are expressed as percentages, with higher values closer to 100% indicating superior efficacy. Heterogeneity analysis of the included studies was performed by drawing forest plots, where square points represent the effect size of each study and horizontal lines represent confidence intervals. Limited or no overlap between studies suggests potential heterogeneity. League tables were created to compare the effects of various interventions. Funnel plots were used to evaluate publication bias.

### 2.5 Sensitivity analysis

To assess robustness, we excluded six studies with high risk of bias (5–10) and re-estimated the network meta-analysis for each outcome. The low-risk model was compared with the full model in terms of effect direction, statistical significance, and between-study heterogeneity (Tau^2^).

## 3 Results

### 3.1 Characteristics and quality of included studies

**FIGURE 1:**
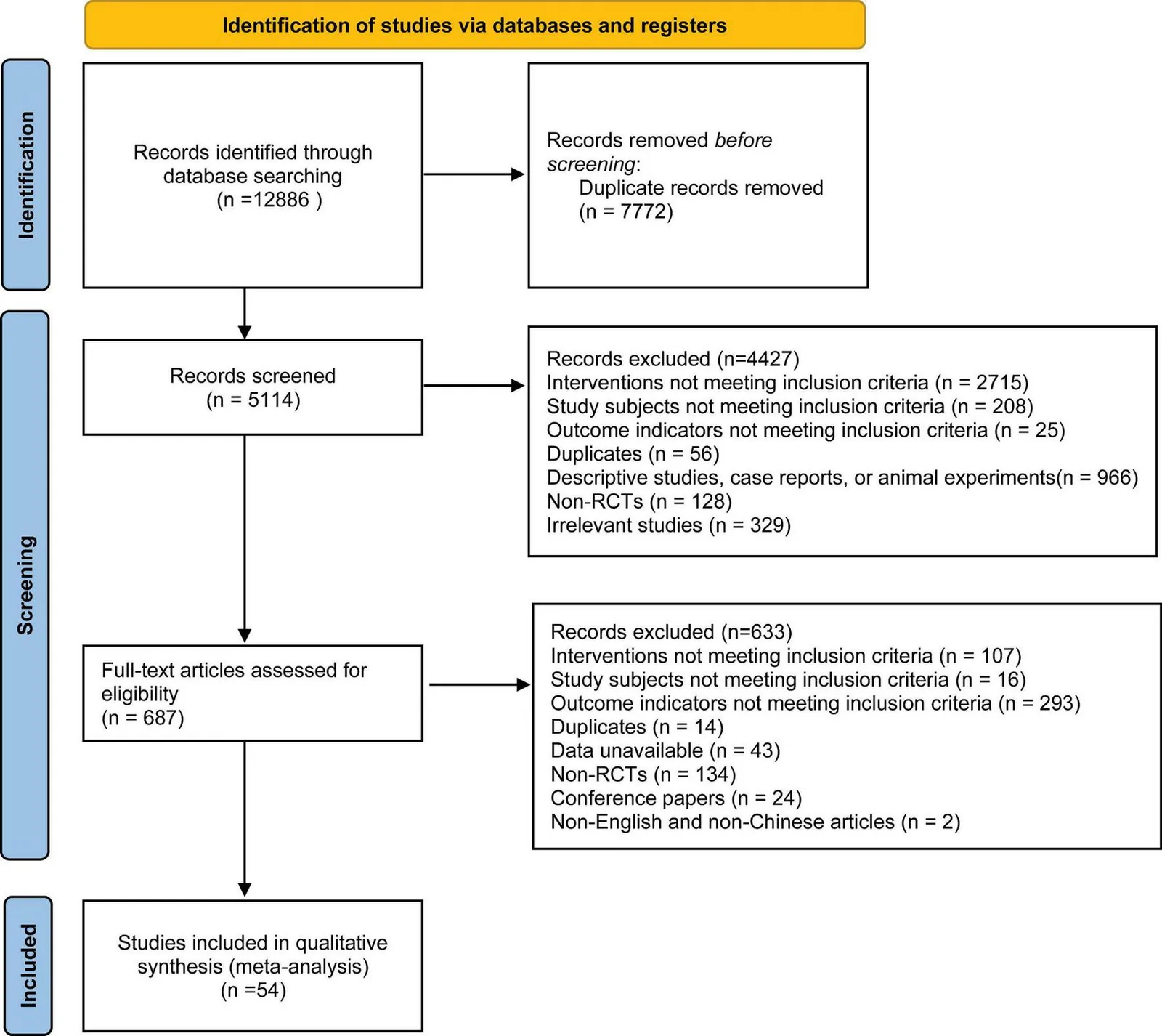
Preferred reporting items for systematic reviews and meta-analyses flow diagram of included and excluded studies.

**FIGURE 2:**
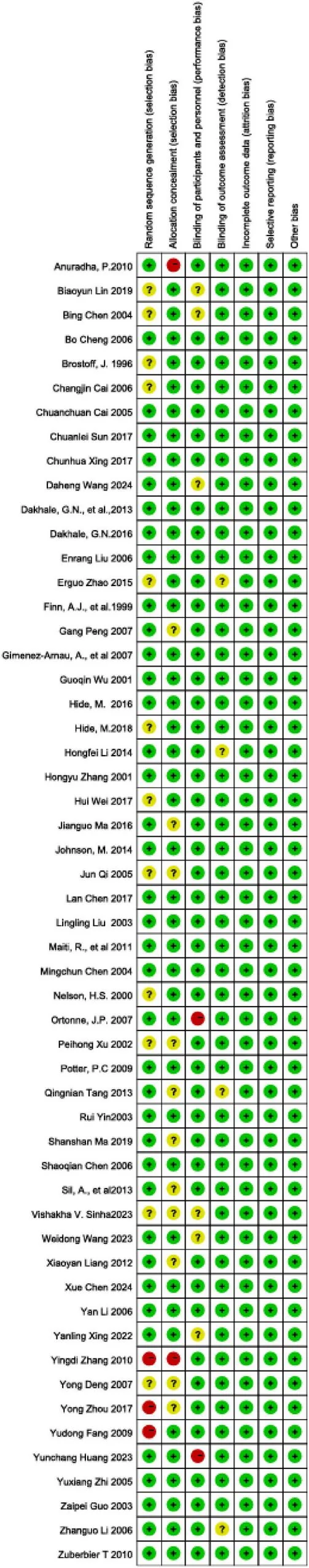
Quality assessment of included studies using the revised Cochrane risk-of-bias tool for randomized trials (RoB 2).

**FIGURE 3:**
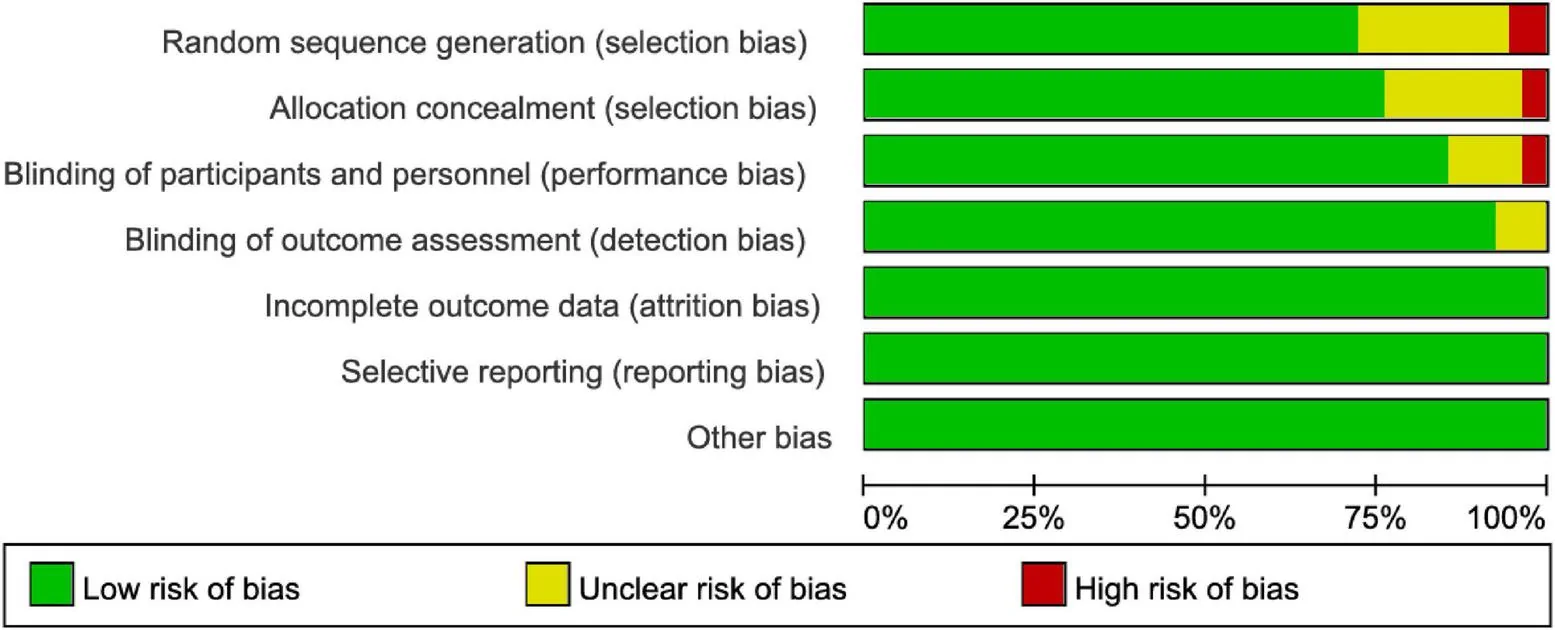
Summarized proportions for each domain of the risk-of-bias assessment from included studies.

Details of the systematic literature search are shown (Figure 1). After screening titles and abstracts and evaluating the studies according to the inclusion/exclusion criteria, 54 randomized controlled trials (RCTs) involving 7,290 patients were included. Of these, 36 reported on TSS, 31 on itch scores, and 27 on wheal scores. Adverse event data were reported in 52 studies. Ten different medications were represented across the included studies, all of which demonstrated comparable baseline characteristics. Baseline characteristics of included studies are presented in Table 1. Regarding study quality, 6 studies were assessed as high risk of bias, 23 as with the valid judgment for the tool used (RoB 2 uses ‘some concerns’), and 25 as low risk of bias (Figure 2, 3).

**TABLE 1:** Characteristics of included studies.

Study	Site of study	Sample size	Intervention	Drug dosage	Treatment time	Male/Female	Type of RCT	Outcome indicator
Finn et al. (18)	USA	86	Fexofenadine	120 mgOD	4w	22/64	Double-blind	B D
		90	Placebo			20/70		
Dakhale et al. (19)	India	31	Cetirizine	10 mgOD 10 mgOD	6w	11/20	Double-blind	A B C D
		33	Rupatadine			13/20		
Dakhale et al. (20)	India	28	Olopatadine	10 mgOD 10 mgOD	6w	12/16	Double-blind	A B C D
		27	Rupatadine			9/18		
Zuberbier et al. (21)	Argentina, Belgium, France, Germany, Poland, Romania, Spain	172	Bilastine	20 mgOD 5 mgOD	28d	63/109	Double-blind	A B C D
		163	Levocetirizine			54/109		
		181	Placebo			40/141		
Potter et al. (22)	Germany, UK	438	Levocetirizine	5 mgOD 5 mgOD	5w	154/284	Double-blind	B C D
		448	Desloratadine			143/306		
Hide et al. (23)	Japan	91	Rupatadine	10 mgOD	2w	32/59	Double-blind	A D
		94	Placebo			37/57		
Ortonne et al. (6)	France	65	Desloratadine	5 mgOD	6w	24/41	Double-blind	A D
		72	Placebo			29/43		
Brostoff et al. (24)	UK	28	Mizolastine	10 mgOD	28d	10/18	Double-blind	A D
		28	Placebo			15/13		
Nelson et al. (25)	USA	90	Fexofenadine	120mg OD	4w	NR	Double-blind	A D
		79	Placebo			NR		
Hide et al. (26)	Japan	100	Bilastine	20 mgOD	2w	35/65	Double-blind	A B C D
		95	Placebo			26/69		
Johnson et al. (27)	India	50	Levocetirizine	10 mgOD	6w	NR	Open	A B C D
		50	Rupatadine			NR		
Anuradha et al. (5)	India	30	Loratadine	10 mgOD 5 mgOD	4w	12/18	NR	A D
		30	Levocetirizine			13/17		
Sil et al. (28)	India	54	Olopatadine	10 mgOD 5 mgOD	5w	38/16	Single-blind	A D
		51	Levocetirizine			29/22		
Maiti et al. (29)	India	26	Rupatadine	10 mgOD	NR	11/15	Single-blind	A B C D
		28	Loratadine			12/16		
Gimenez-Arnau et al. (30)	Spain	110	Rupatadine	10 mgOD	4w	33/77	Double-blind	B D
		111	Placebo			42/69		
Enrang et al. (31)	China	44	Desloratadine	5 mgOD	14d	35/52	NR	A B C D
		43	Cetirizine			10 mgOD		
Chen et al. (32)	China	49	Desloratadine	5 mgOD 10 mgOD	14d	NR	NR	B C D
		45	Mizolastine			NR		
Ma (33)	China	32	Desloratadine	5 mgOD 10 mgOD	30d	19/13	NR	A D
		32	Mizolastine			11/21		
Tang and Mo (34)	China	58	Desloratadine	5 mgOD 10 mgOD	14d	34/24	NR	A D
		58	Mizolastine			36/22		
Liang (35)	China	61	Desloratadine	5 mgOD	30d	78/44	NR	A D
		61	Mizolastine			10 mgOD		
Li (36)	China	40	Desloratadine	5 mgOD 10 mgOD	14d	18/22	NR	A D
		40	Cetirizine			19/21		
Li et al. (37)	China	45	Desloratadine	5 mgOD 10 mgOD	28d	26/19	NR	B C D
		33	Cetirizine			17/16		
Mingchun et al. (38)	China	60	Fexofenadine	180 mgOD 10 mgOD	14d	21/39	Double-blind	A D
		62	Loratadine			31/31		
Xing et al. (39)	China	72	Rupatadine	10 mgOD 10 mgOD	28d	NR	Double-blind	A D
		72	Cetirizine			NR		
Sun (40)	China	37	Rupatadine	10 mgOD 10 mgOD	4w	13/24	NR	A D
		38	Loratadine			12/26		
Cai et al. (41)	China	85	Mizolastine	10 mgOD 5 mgOD	28d	NR	Double-blind	B C D
		85	Levocetirizine			NR		
Xu and Yuan (42)	China	50	Mizolastine	10 mgOD 10 mgOD	28d	19/31	NR	A D
		30	Loratadine			14/16		
Zhang et al. (43)	China	71	Mizolastine	10 mgOD 10 mgOD	28d	27/44	NR	B C D
		74	Loratadine			19/55		
Guo et al. (44)	China	23	Mizolastine	10 mgOD 10 mgOD	28d	11/12	Double-blind	A D
		23	Loratadine			9/14		
Wu et al. (45)	China	29	Mizolastine	10 mgOD 10 mgOD	28d	NR	Open	B C D
		31	Loratadine			NR		
Liu et al. (46)	China	108	Mizolastine	10 mgOD 10 mgOD	28d	46/62	Double-blind	B C D
		105	Loratadine			50/55		
Chen et al. (47)	China	72	Olopatadine	10 mgOD 10 mgOD	4w	NR	Double-blind	A D
		71	Loratadine			NR		
Ma and Zhang (48)	China	59	Olopatadine	10 mgOD 5 mgOD	4w	25/34	NR	A D
		59	Levocetirizine			23/36		
Wei (49)	China	81	Olopatadine	10 mgOD	28d	45/36	NR	C D
		81	Loratadine			44/37		
Li et al. (50)	China	67	Fexofenadine	120 mgOD 10 mgOD	2w	NR	Double-blind	A D
		69	Cetirizine			NR		
Zhao et al. (51)	China	60	Levocetirizine	5 mgOD 10 mgOD	4w	23/37	NR	A D
		60	Loratadine			28/32		
Cheng et al. (52)	China	15	Levocetirizine	5 mgOD 10mg OD	2w	NR	Double-blind	B C D
		18	Cetirizine			NR		
Fang et al. (9)	China	48	Levocetirizine	5 mgOD 10 mgOD	2w	16/32	NR	A D
		48	Cetirizine			20/28		
Yin et al. (53)	China	22	Levocetirizine	5 mgOD 10 mgOD	28d	10/12	Double-blind	A B C D
		22	Cetirizine			8/14		
Zhang and Wang (7)	China	54	Levocetirizine	5 mgOD 10 mgOD	28d	29/25	NR	A B C D
		52	Cetirizine			27/25		
Cai and Chen (54)	China	60	Ebastine	10 mgOD 10 mgOD	28d	NR	Double-blind	A B C D
		60	Mizolastine			NR		
Xiang et al. (55)	China	69	Ebastine	10 mgOD 10 mgOD	14d	NR	Double-blind	A B C D
		66	Cetirizine			NR		
Lin (56)	China	41	Ebastine	10 mgOD 10 mgOD	28d	16/25	NR	A B C D
		41	Loratadine			17/24		
Deng et al. (57)	China	82	Ebastine	10 mgOD 10 mgOD	28d	NR	NR	A B C D
		80	Loratadine			NR		
Qi et al. (58)	China	90	Ebastine	10 mgOD 10 mgOD	14d	NR	Double-blind	A D
		98	Loratadine		NR		
Zhou (8)	China	68	Levocetirizine	5 mgOD 10 mgOD	4w	NR	NR	A D
		68	Cetirizine			NR		
Gang (59)	China	30	Mizolastine	10 mgOD 10 mgOD	28d	12/18	Open	B C D
		32	Loratadine			12/20		
Chen et al. (60)	China	93	Ebastine	10 mgOD 5 mgOD	28d	NR	Double-blind	A B C D
		90	Levocetirizine			NR		
Wang (61)	China	30	Ebastine	10 mgOD 5 mgOD	14d 14d	14/16	NR	D
		30	Desloratadine			12/18		
Xing (62)	China	37	Levocetirizine	5 mgOD 5 mgOD	4w 4w	20/17	NR	D
		37	Desloratadine			18/19		
Yunchang (10)	China	45	Rupatadine	10 mgOD 5 mgOD	7d 7d	23/22	NR	B C D
		45	Levocetirizine			28/17		
Wang (63)	China	30	Rupatadine	10 mgOD 5 mgOD	3w 3w	14/16	NR	AD
		30	Levocetirizine			15/15		
Chen et al. (64)	China	122	Bilastine	20 mgOD 5 mgOD	28d 28d	NR	Double-blind	A B C
		120	Levocetirizine			NR		
Vishakha et al. (65)	India	32	Bilastine	20 mgOD 10 mgOD	6w 6w	13/19	NR	A B C
		31	Cetirizine			13/18		

A, TSS, total symptom score; B, itch score; C, wheal score; D, adverse reaction rate.

### 3.2 Changes in TSS from baseline

**FIGURE 4:**
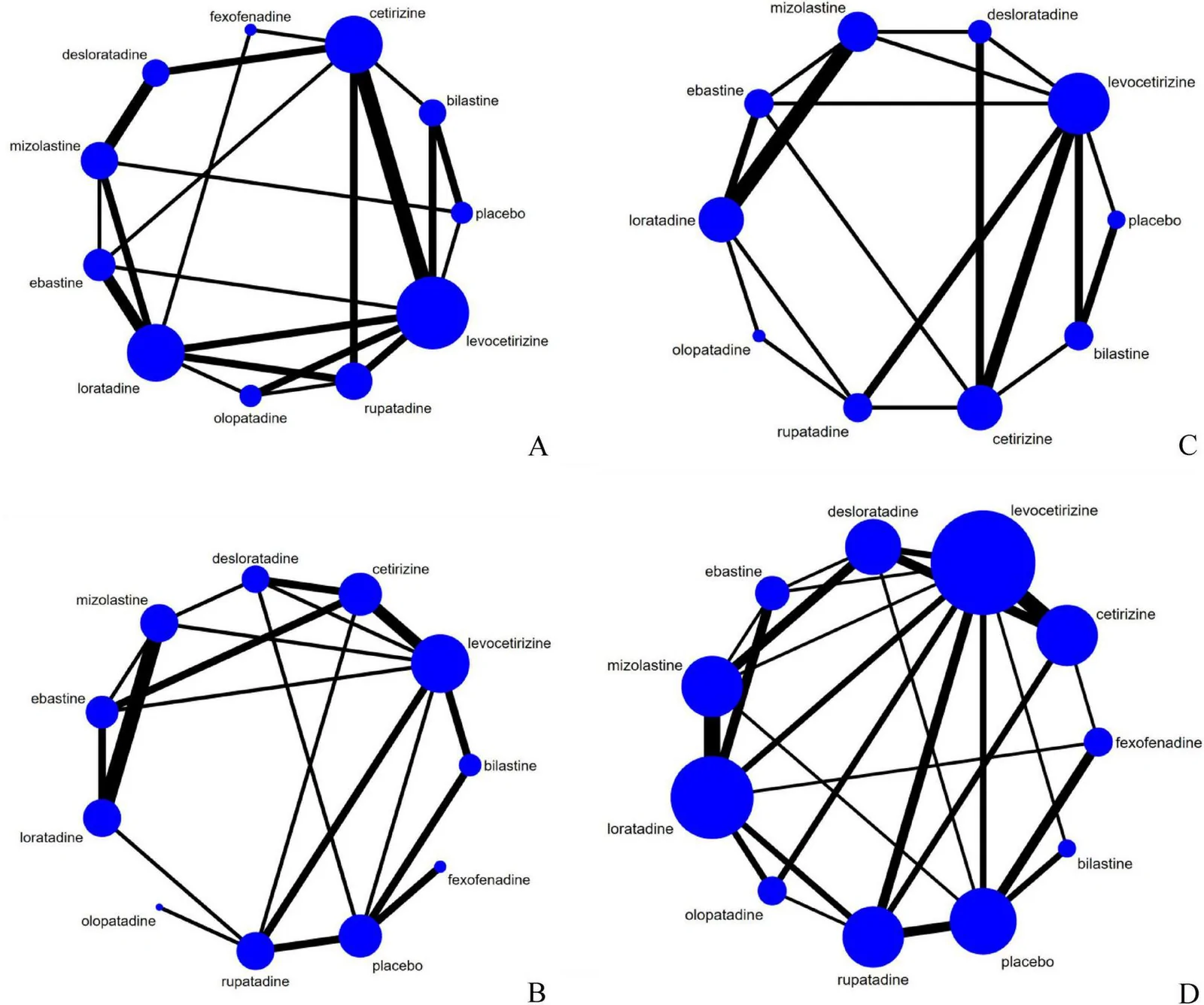
Networks of all treatment comparisons according to the outcomes measured. **(A)** Total symptom score changes from baseline. **(B)** Pruritus score changes from baseline. **(C)** Wheal score changes from baseline. **(D)** Adverse reaction rate. The size of the nodes corresponds to the number of treatment trials. Treatment pairs with direct evidence are connected with solid black lines. The thickness of these lines corresponds to the number of participants within each comparison.

**FIGURE 5:**
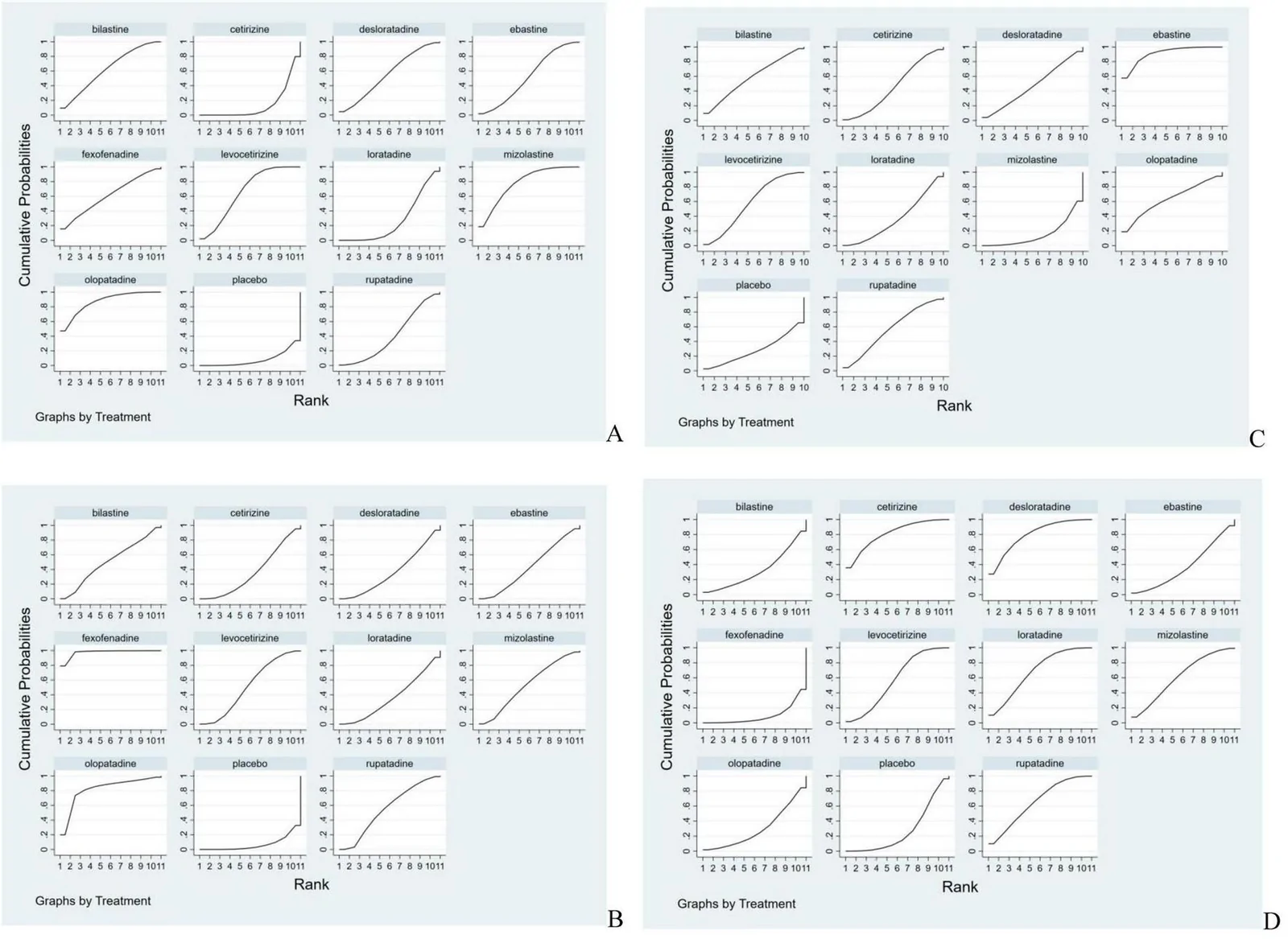
SUCRA ranking curves according to the outcomes measured. **(A)** Total symptom score changes from baseline. **(B)** Pruritus score changes from baseline. **(C)** Wheal score changes from baseline. **(D)** Adverse reaction rate.

Figure 4 and Table 2 present the efficacy of therapeutic interventions on TSS changes across 36 included studies (4,275 patients, 10 treatment drugs). All included sgAHs demonstrated superior TSS improvement compared to placebo. However, only four drugs showed statistically significant differences. Olopatadine showed significantly greater efficacy than placebo (SMD 0.97 [95% CI: 0.19–1.75]). In contrast, mizolastine, levocetirizine, and bilastine showed moderate efficacy, with standardized mean differences (SMD 0.83 [95% CI: 0.15–1.51], 0.70 [95% CI: 0.08–1.31] and 0.67 [95% CI: 0.09–1.25] respectively). Olopatadine demonstrated statistically superior efficacy compared to cetirizine and loratadine. Mizolastine and levocetirizine also showed statistically superior efficacy compared to cetirizine. Based on the surface under the cumulative ranking curve (SUCRA), olopatadine ranked highest, followed by mizolastine and levocetirizine (Figure 5).

**TABLE 2:** League table of standardized mean difference (SMD) for total symptom score (TSS) changes from baseline in patients with CU.

Treatment										
OLOP	0.15(−0.54, 0.84)	0.28 (−0.23, 0.79)	0.30 (−0.41, 1.02)	0.30 (−0.55, 1.15)	0.36 (−0.37, 1.10)	0.40 (−0.24, 1.04)	0.51 (−0.07, 1.08)	**0.62 (0.07, 1.18)**	**0.77 (0.19, 1.36)**	**0.97 (0.19, 1.75)**
−0.15 (−0.84, 0.54)	MIZO	0.13 (−0.40, 0.67)	0.16 (−0.52, 0.83)	0.15 (−0.66, 0.96)	0.22 (−0.27, 0.70)	0.25 (−0.30, 0.79)	0.36 (−0.25, 0.96)	0.48 (−0.02, 0.98)	**0.63 (0.10, 1.16)**	**0.83 (0.15, 1.51)**
−0.28 (−0.79, 0.23)	−0.13 (−0.67, 0.40)	LEVO	0.03 (−0.50, 0.55)	0.02 (−0.72, 0.76)	0.09 (−0.50, 0.67)	0.12 (−0.36, 0.59)	0.23 (−0.20, 0.66)	0.35 (−0.05, 0.75)	**0.50 (0.13, 0.86)**	**0.70 (0.08, 1.31)**
−0.30 (−1.02, 0.41)	−0.16 (−0.83, 0.52)	−0.03 (−0.55, 0.50)	BILA	−0.01 (−0.87, 0.86)	0.06 (−0.66, 0.78)	0.09 (−0.57, 0.76)	0.20 (−0.44, 0.85)	0.32 (−0.30, 0.94)	0.47 (−0.09, 1.04)	**0.67 (0.09, 1.25)**
−0.30 (−1.15, 0.55)	−0.15 (−0.96, 0.66)	−0.02 (−0.76, 0.72)	0.01 (−0.86, 0.87)	FEXO	0.07 (−0.78, 0.91)	0.10 (−0.68, 0.88)	0.21 (−0.57, 0.99)	0.33 (−0.38, 1.03)	0.48 (−0.22, 1.18)	0.68 (−0.24, 1.59)
−0.36 (−1.10, 0.37)	−0.22 (−0.70, 0.27)	−0.09 (−0.67, 0.50)	−0.06 (−0.78, 0.66)	−0.07 (−0.91, 0.78)	DESL	0.03 (−0.60, 0.66)	0.14 (−0.51, 0.79)	0.26 (−0.33, 0.85)	0.41 (−0.13, 0.95)	0.61 (−0.15, 1.37)
−0.40 (−1.04, 0.24)	−0.25 (−0.79, 0.30)	−0.12 (−0.59, 0.36)	−0.09 (−0.76, 0.57)	−0.10 (−0.88, 0.68)	−0.03 (−0.66, 0.60)	EBAS	0.11 (−0.45, 0.66)	0.23 (−0.20, 0.65)	0.38 (−0.12, 0.87)	0.58 (−0.14, 1.30)
−0.51 (−1.08, 0.07)	−0.36 (−0.96, 0.25)	−0.23 (−0.66, 0.20)	−0.20 (−0.85, 0.44)	−0.21 (−0.99, 0.57)	−0.14 (−0.79, 0.51)	−0.11 (−0.66, 0.45)	RUPA	0.12 (−0.34, 0.58)	0.27 (−0.18, 0.72)	0.47 (−0.25, 1.18)
−0.62 (−1.18, −0.07)	−0.48 (−0.98, 0.02)	−0.35 (−0.75, 0.05)	−0.32 (−0.94, 0.30)	−0.33 (−1.03, 0.38)	−0.26 (−0.85, 0.33)	−0.23 (−0.65, 0.20)	−0.12 (−0.58, 0.34)	LORA	0.15 (−0.29, 0.59)	0.35 (−0.33, 1.03)
−0.77 (−1.36, −0.19)	−0.63 (−1.16, −0.10)	−0.50 (−0.86, −0.13)	−0.47 (−1.04, 0.09)	−0.48 (−1.18, 0.22)	−0.41 (−0.95, 0.13)	−0.38 (−0.87, 0.12)	−0.27 (−0.72, 0.18)	−0.15 (−0.59, 0.29)	CETI	0.20 (−0.46, 0.85)
−0.97 (−1.75, −0.19)	−0.83 (−1.51, −0.15)	−0.70 (−1.31, −0.08)	−0.67 (−1.25, −0.09)	−0.68 (−1.59, 0.24)	−0.61 (−1.37, 0.15)	−0.58 (−1.30, 0.14)	−0.47 (−1.18, 0.25)	−0.35 (−1.03, 0.33)	−0.20 (−0.85, 0.46)	PLAC

TSS, Total symptom score; SMD, standardized mean difference; 95% CI, 95% confidence interval. Statistical significance is indicated by boldface type. BILA, Bilastine; CETI, cetirizine; DESL, desloratadine; EBAS, ebastine; FEXO, fexofenadine; LEVO, levocetirizine; LORA, loratadine; MIZO, mizolastine; OLOP, olopatadine; PLAC, placebo; RUPA, rupatadine. Boldface indicates statistical significance (95% CI excludes the null value: 0 for SMD and 1 for OR).

### 3.3 Changes in pruritus score from baseline

Figure 4 and Table 3 present the overall results of itch score changes across 31 included studies (4,993 patients, 10 interventions). Pairwise comparisons of the 10 drugs against placebo revealed that fexofenadine and rupatadine significantly reduced itch scores (standardized mean difference (SMD: 2.56 [95% CI: 1.48–3.63] and 0.82 [95% CI: 0.02–1.61], respectively). Fexofenadine exhibited the largest effect size, while rupatadine demonstrated a moderate but statistically significant improvement. While the remaining eight drugs showed numerically greater reductions in itch scores compared to placebo, these differences were not statistically significant. Furthermore, fexofenadine demonstrated significantly greater pruritus score reduction than rupatadine, mizolastine, levocetirizine, bilastine, ebastine, desloratadine, cetirizine, and loratadine. The probability of pruritus score reduction, calculated using SUCRA, ranked fexofenadine, olopatadine, and rupatadine highest, with placebo ranking last (Figure 5).

**TABLE 3:** League table of standardized mean difference (SMD) for pruritus score changes from baseline in patients with CU.

Treatment										
FEXO	0.84 (−1.19, 2.88)	**1.74 (0.40, 3.07)**	**1.77 (0.28, 3.27)**	**1.80 (0.47, 3.14)**	**1.79 (0.40, 3.19)**	**1.90 (0.41, 3.39)**	**1.99 (0.58, 3.39)**	**2.00 (0.58, 3.41)**	**1.99 (0.48, 3.50)**	**2.56 (1.48, 3.63)**
−0.84 (−2.88, 1.19)	OLOP	0.89 (−0.65, 2.43)	0.93 (−0.88, 2.74)	0.96 (−0.74, 2.66)	0.95 (−0.90, 2.80)	1.06 (−0.74, 2.86)	1.14 (−0.60, 2.88)	1.15 (−0.64, 2.95)	1.14 (−0.66, 2.95)	1.71 (−0.02, 3.45)
-1.74 (−3.07, −0.40)	−0.89 (−2.43, 0.65)	RUPA	0.04 (−0.92, 0.99)	0.06 (−0.65, 0.78)	0.05 (−0.97, 1.08)	0.16 (−0.77, 1.10)	0.25 (−0.57, 1.06)	0.26 (−0.66, 1.19)	0.25 (−0.69, 1.19)	**0.82 (0.02, 1.61)**
-1.77 (−3.27, −0.28)	−0.93 (−2.74, 0.88)	−0.04 (−0.99, 0.92)	MIZO	0.03 (−0.80, 0.86)	0.02 (−1.15, 1.19)	0.13 (−0.67, 0.93)	0.21 (−0.67, 1.09)	0.22 (−0.70, 1.15)	0.21 (−0.43, 0.86)	0.78 (−0.26, 1.83)
-1.80 (−3.14, −0.47)	−0.96 (−2.66, 0.74)	−0.06 (−0.78, 0.65)	−0.03 (−0.86, 0.80)	LEVO	−0.01 (−0.90, 0.88)	0.10 (−0.69, 0.89)	0.18 (−0.46, 0.83)	0.20 (−0.58, 0.98)	0.19 (−0.68, 1.06)	0.75 (−0.04, 1.55)
-1.79 (−3.19, −0.40)	−0.95 (−2.80, 0.90)	−0.05 (−1.08, 0.97)	−0.02 (−1.19, 1.15)	0.01 (−0.88, 0.90)	BILA	0.11 (−1.04, 1.26)	0.19 (−0.86, 1.24)	0.21 (−0.89, 1.30)	0.20 (−0.99, 1.39)	0.76 (−0.13, 1.66)
-1.90 (−3.39, −0.41)	−1.06 (−2.86, 0.74)	−0.16 (−1.10, 0.77)	−0.13 (−0.93, 0.67)	−0.10 (−0.89, 0.69)	−0.11 (−1.26, 1.04)	EBAS	0.08 (−0.69, 0.85)	0.10 (−0.84, 1.04)	0.09 (−0.69, 0.86)	0.65 (−0.38, 1.69)
-1.99 (−3.39, −0.58)	−1.14 (−2.88, 0.60)	−0.25 (−1.06, 0.57)	−0.21 (−1.09, 0.67)	−0.18 (−0.83, 0.46)	−0.19 (−1.24, 0.86)	−0.08 (−0.85, 0.69)	CETI	0.01 (−0.76, 0.78)	0.00 (−0.90, 0.91)	0.57 (−0.34, 1.49)
-2.00 (−3.41, −0.58)	−1.15 (−2.95, 0.64)	−0.26 (−1.19, 0.66)	−0.22 (−1.15, 0.70)	−0.20 (−0.98, 0.58)	−0.21 (−1.30, 0.89)	−0.10 (−1.04, 0.84)	−0.01 (−0.78, 0.76)	DESL	−0.01 (−0.99, 0.97)	0.56 (−0.37, 1.48)
-1.99 (−3.50, −0.48)	−1.14 (−2.95, 0.66)	−0.25 (−1.19, 0.69)	−0.21 (−0.86, 0.43)	−0.19 (−1.06, 0.68)	−0.20 (−1.39, 0.99)	−0.09 (−0.86, 0.69)	−0.00 (−0.91, 0.90)	0.01 (−0.97, 0.99)	LORA	0.57 (−0.50, 1.63)
-2.56 (−3.63, −1.48)	−1.71 (−3.45, 0.02)	−0.82 (−1.61, −0.02)	−0.78 (−1.83, 0.26)	−0.75 (−1.55, 0.04)	−0.76 (−1.66, 0.13)	−0.65 (−1.69, 0.38)	−0.57 (−1.49, 0.34)	−0.56 (−1.48, 0.37)	−0.57 (−1.63, 0.50)	PLAC

SMD, standardized mean difference; 95% CI, 95% confidence interval. Statistical significance is indicated by boldface type. BILA, Bilastine; CETI, cetirizine; DESL, desloratadine; EBAS, ebastine; FEXO, fexofenadine; LEVO, levocetirizine; LORA, loratadine; MIZO, mizolastine; OLOP, olopatadine; PLAC, placebo; RUPA, rupatadine. Boldface indicates statistical significance (95% CI excludes the null value: 0 for SMD and 1 for OR).

### 3.4 Changes in wheal score from baseline

Twenty-seven studies evaluating the efficacy of nine drugs on urticarial wheal reduction were included, encompassing a total of 4,201 participants (Figure 4 and Table 4). All drugs except mizolastine showed greater efficacy than placebo, though these differences were not statistically significant. Ebastine was signif-icantly and statistically superior to loratadine (SMD 1.27 95% CI: [0.15–2.39]) and mizolastine (SMD 1.74 95% CI: [0.54–2.93]). According to the SUCRA numerical ranking of the nine drugs and placebo in terms of improvement in wheal scores, the top three were ebastine, olopatadine, and bilastine. Of note, mizolastine ranked tenth, performing below placebo (Figure 5).

**TABLE 4:** League table of standardized mean difference (SMD) for wheal score changes from baseline in patients with CU.

Treatment									
EBAS	0.67 (−1.20, 2.53)	0.85 (−0.81, 2.50)	0.87 (−0.34, 2.09)	0.87 (−0.55, 2.29)	1.06 (−0.44, 2.56)	1.06 (−0.20, 2.33)	**1.27 (0.15, 2.39)**	1.54 (−0.45, 3.54)	**1.74 (0.54, 2.93)**
−0.67 (−2.53, 1.20)	OLOP	0.18 (−1.95, 2.31)	0.21 (−1.59, 2.01)	0.21 (−1.44, 1.85)	0.39 (−1.63, 2.41)	0.40 (−1.47, 2.26)	0.61 (−1.03, 2.24)	0.88 (−1.52, 3.28)	1.07 (−0.72, 2.86)
−0.85 (−2.50, 0.81)	−0.18 (−2.31, 1.95)	BILA	0.03 (−1.19, 1.25)	0.03 (−1.58, 1.63)	0.21 (−1.42, 1.84)	0.22 (−1.12, 1.56)	0.43 (−1.25, 2.10)	0.70 (−0.72, 2.12)	0.89 (−0.76, 2.54)
−0.87 (−2.09, 0.34)	−0.21 (−2.01, 1.59)	−0.03 (−1.25, 1.19)	LEVO	−0.00 (−1.12, 1.12)	0.18 (−1.03, 1.40)	0.19 (−0.70, 1.09)	0.40 (−0.82, 1.62)	0.67 (−0.96, 2.30)	0.86 (−0.33, 2.06)
−0.87 (−2.29, 0.55)	−0.21 (−1.85, 1.44)	−0.03 (−1.63, 1.58)	0.00 (−1.12, 1.12)	RUPA	0.18 (−1.32, 1.69)	0.19 (−1.04, 1.42)	0.40 (−0.88, 1.68)	0.67 (−1.27, 2.62)	0.87 (−0.50, 2.23)
-1.06 (−2.56, 0.44)	−0.39 (−2.41, 1.63)	−0.21 (−1.84, 1.42)	−0.18 (−1.40, 1.03)	−0.18 (−1.69, 1.32)	DESL	0.01 (−1.15, 1.17)	0.22 (−1.26, 1.69)	0.49 (−1.49, 2.47)	0.68 (−0.70, 2.06)
-1.06 (−2.33, 0.20)	−0.40 (−2.26, 1.47)	−0.22 (−1.56, 1.12)	−0.19 (−1.09, 0.70)	−0.19 (−1.42, 1.04)	−0.01 (−1.17, 1.15)	CETI	0.21 (−1.10, 1.52)	0.48 (−1.28, 2.24)	0.67 (−0.61, 1.96)
-1.27 (−2.39, −0.15)	−0.61 (−2.24, 1.03)	−0.43 (−2.10, 1.25)	−0.40 (−1.62, 0.82)	−0.40 (−1.68, 0.88)	−0.22 (−1.69, 1.26)	−0.21 (−1.52, 1.10)	LORA	0.27 (−1.73, 2.27)	0.47 (−0.45, 1.39)
-1.54 (−3.54, 0.45)	−0.88 (−3.28, 1.52)	−0.70 (−2.12, 0.72)	−0.67 (−2.30, 0.96)	−0.67 (−2.62, 1.27)	−0.49 (−2.47, 1.49)	−0.48 (−2.24, 1.28)	−0.27 (−2.27, 1.73)	PLAC	0.19 (−1.79, 2.18)
-1.74 (−2.93, −0.54)	−1.07 (−2.86, 0.72)	−0.89 (−2.54, 0.76)	−0.86 (−2.06, 0.33)	−0.87 (−2.23, 0.50)	−0.68 (−2.06, 0.70)	−0.67 (−1.96, 0.61)	−0.47 (−1.39, 0.45)	−0.19 (−2.18, 1.79)	MIZO

SMD, standardized mean difference; 95% CI, 95% confidence interval. Statistical significance is indicated by boldface type. BILA, Bilastine; CETI, cetirizine; DESL, desloratadine; EBAS, ebastine; LEVO, levocetirizine; LORA, loratadine; MIZO, mizolastine; OLOP, olopatadine; PLAC, placebo; RUPA, rupatadine. Boldface indicates statistical significance (95% CI excludes the null value: 0 for SMD and 1 for OR).

### 3.5 Incidence of adverse reactions

A summary of 52 studies with 3,782 patients assessed for the acceptability of treatments is shown in Figure 4 and Table 5. Desloratadine and cetirizine had significantly higher adverse event rates than fexofenadine (OR 2.35 [95% CI: 1.14–4.86] and 2.30 [95% CI: 1.09–4.83], respectively). Except for fexofenadine, the other nine drugs had higher adverse event rates than placebo. Treatment acceptability, ranked by SUCRA values, is shown in Figure 5.

**TABLE 5:** Adverse reaction rate from baseline in patients with CU.

Treatment										
CETI	0.98 (0.52, 1.82)	0.85 (0.43, 1.68)	0.82 (0.43, 1.58)	0.81 (0.40, 1.64)	0.77 (0.43, 1.36)	0.64 (0.29, 1.39)	0.58 (0.25, 1.37)	0.58 (0.26, 1.31)	0.57 (0.30, 1.09)	0.43 (0.21, 0.88)
1.02 (0.55, 1.91)	DESL	0.87 (0.47, 1.60)	0.84 (0.44, 1.62)	0.83 (0.46, 1.49)	0.79 (0.46, 1.33)	0.65 (0.33, 1.31)	0.60 (0.26, 1.37)	0.60 (0.28, 1.29)	0.59 (0.32, 1.09)	0.44 (0.21, 0.92)
1.18 (0.60, 2.33)	1.15 (0.62, 2.12)	LORA	0.97 (0.55, 1.71)	0.95 (0.59, 1.53)	0.91 (0.56, 1.47)	0.75 (0.42, 1.34)	0.69 (0.31, 1.53)	0.69 (0.37, 1.28)	0.68 (0.38, 1.20)	0.50 (0.25, 1.02)
1.21 (0.63, 2.33)	1.18 (0.62, 2.27)	1.03 (0.58, 1.81)	RUPA	0.98 (0.52, 1.85)	0.93 (0.56, 1.54)	0.78 (0.38, 1.59)	0.71 (0.33, 1.52)	0.71 (0.35, 1.43)	0.70 (0.43, 1.14)	0.52 (0.26, 1.03)
1.24 (0.61, 2.52)	1.21 (0.67, 2.19)	1.05 (0.65, 1.69)	1.02 (0.54, 1.94)	MIZO	0.95 (0.56, 1.62)	0.79 (0.42, 1.49)	0.72 (0.31, 1.66)	0.72 (0.35, 1.49)	0.71 (0.39, 1.32)	0.53 (0.25, 1.12)
1.30 (0.73, 2.31)	1.27 (0.75, 2.15)	1.10 (0.68, 1.79)	1.07 (0.65, 1.77)	1.05 (0.62, 1.79)	LEVO	0.83 (0.45, 1.53)	0.76 (0.38, 1.52)	0.76 (0.40, 1.44)	0.75 (0.47, 1.19)	0.55 (0.29, 1.07)
1.56 (0.72, 3.41)	1.53 (0.76, 3.05)	1.33 (0.74, 2.37)	1.29 (0.63, 2.64)	1.26 (0.67, 2.37)	1.20 (0.66, 2.20)	EBAS	0.91 (0.37, 2.22)	0.91 (0.41, 2.01)	0.90 (0.44, 1.82)	0.67 (0.29, 1.53)
1.72 (0.73, 4.05)	1.68 (0.73, 3.87)	1.46 (0.66, 3.25)	1.42 (0.66, 3.06)	1.39 (0.60, 3.19)	1.32 (0.66, 2.65)	1.10 (0.45, 2.69)	BILA	1.00 (0.40, 2.49)	0.99 (0.52, 1.88)	0.73 (0.32, 1.69)
1.72 (0.76, 3.88)	1.68 (0.78, 3.64)	1.46 (0.78, 2.72)	1.42 (0.70, 2.87)	1.39 (0.67, 2.87)	1.32 (0.70, 2.50)	1.10 (0.50, 2.43)	1.00 (0.40, 2.49)	OLOP	0.99 (0.48, 2.04)	0.73 (0.31, 1.71)
1.74 (0.92, 3.30)	1.70 (0.92, 3.14)	1.48 (0.83, 2.62)	1.43 (0.88, 2.34)	1.40 (0.76, 2.60)	1.34 (0.84, 2.12)	1.11 (0.55, 2.25)	1.01 (0.53, 1.93)	1.01 (0.49, 2.09)	PLAC	0.74 (0.43, 1.28)
**2.35 (1.14, 4.86)**	**2.30 (1.09, 4.83)**	2.00 (0.98, 4.06)	1.94 (0.97, 3.86)	1.90 (0.89, 4.03)	1.81 (0.94, 3.49)	1.50 (0.65, 3.45)	1.37 (0.59, 3.15)	1.37 (0.58, 3.20)	1.35 (0.78, 2.34)	FEXO

OR, Odds ratio; 95% CI, 95% confidence interval. Statistical significance is indicated by boldface type. BILA, Bilastine; CETI, cetirizine; DESL, desloratadine; EBAS, ebastine; FEXO, fexofenadine; LEVO, levocetirizine; LORA, loratadine; MIZO, mizolastine; OLOP, olopatadine; PLAC, placebo; RUPA, rupatadine. Boldface indicates statistical significance (95% CI excludes the null value: 0 for SMD and 1 for OR).

### 3.6 Assessment of heterogeneity, inconsistency, transitivity, publication bias, and strength of evidence

**FIGURE 6:**
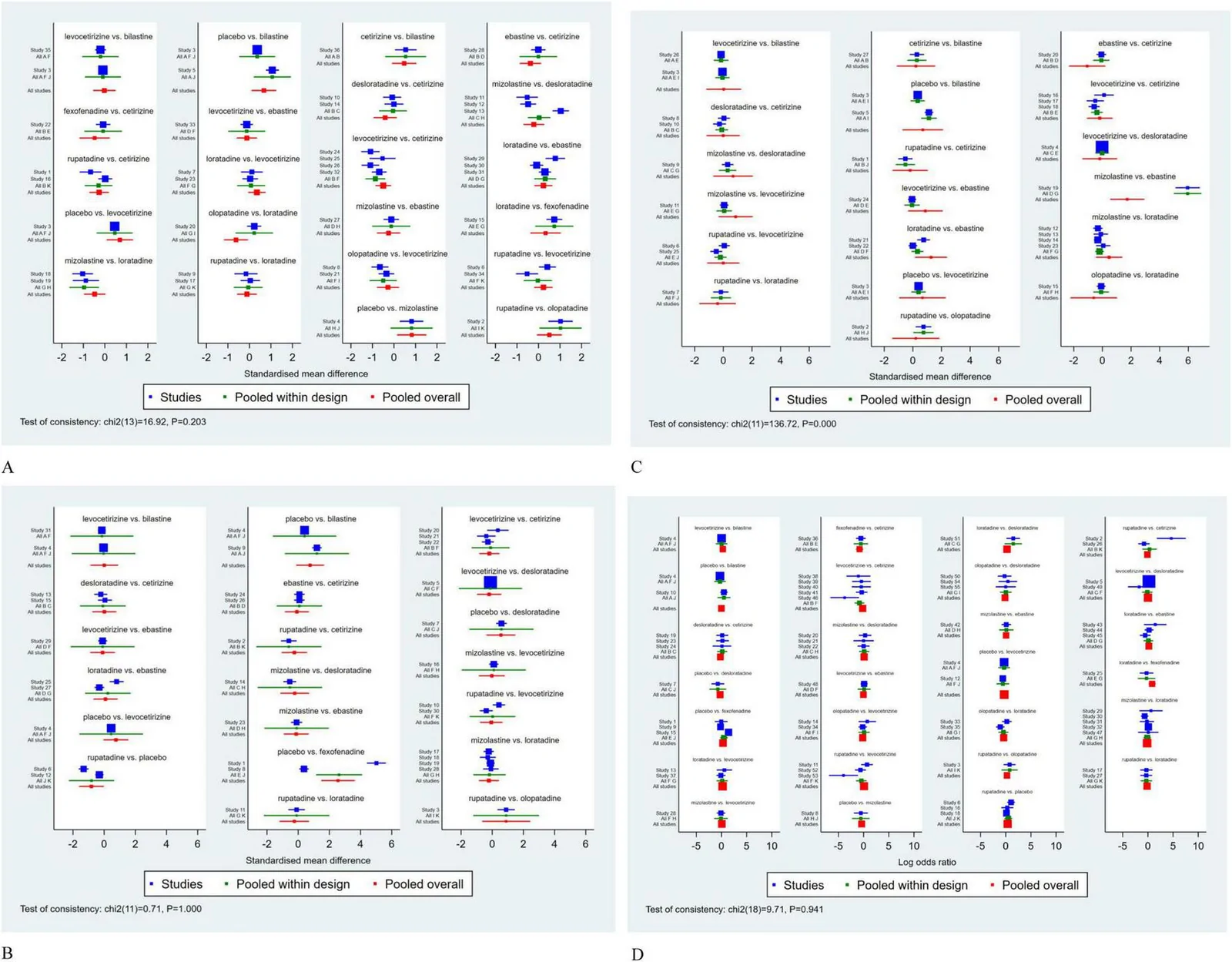
Forest plot showing results of network meta-analysis of all treatment options compared with placebo using randomized controlled trials. **(A)** Total symptom score changes from baseline. **(B)** Pruritus score changes from baseline. **(C)** Wheal score changes from baseline. **(D)** Adverse reaction rate.

Heterogeneity tests were conducted on the included studies, with the following results. The pairwise comparison forest plot is shown in Figure 6. In the figure, square points represent the effect size of each study; larger points indicate greater weight, meaning higher contribution of that study to the meta-analysis. Horizontal lines represent 95% confidence intervals. If there is little or no overlap between study intervals, heterogeneity may exist. Blue, green, and red represent direct comparison, indirect comparison, and mixed comparison effect sizes, respectively. Except for studies on wheal score, the analyses of the other three outcome indicators showed no statistically significant differences between direct and indirect comparisons among medications, indicating stable statistical results with little possibility of inconsistency. The inconsistency test for wheal score studies revealed P < 0.001, indicating inconsistency. Node-splitting analysis of partial node inconsistency found that the overall stability was moderate, possibly due to studies at the ebastine-mizolastine and loratadine-mizolastine nodes.

### 3.7 Sensitivity analysis

To evaluate the robustness of our findings, we excluded studies with high risk of bias and re-estimated the network meta-analysis for all four outcomes. The results demonstrated that the direction and magnitude of treatment effects remained consistent between the full model and the low-risk model across the total symptom score (TSS), pruritus score, wheal score, and adverse events (Supplementary Tables S1–S4).

## 4 Discussion

CU is a common autoimmune dermatosis, characterized by pruritus and wheals, which markedly impair patients’ quality of life. In more than 50% of cases, the disease persists for over 5 years (11). A comprehensive NMA integrating multiple data sources has been lacking. SgAHs are the first-line drugs, but the wide array of products often leads to empirical prescribing and a comprehensive NMA integrating multiple data sources has been lacking (12, 13). Internationally and domestically, many sgAHs are in clinical use, including loratadine, desloratadine, cetirizine, levocetirizine, ebastine, mizolastine, fexofenadine, olopatadine, rupatadine, bilastine, acrivastine and epinastine. Numerous RCTs have confirmed that sgAHs effectively relieve CU symptoms. Recent meta-analyses at Chinese and International have assessed their efficacy, but their data sources and conclusions are inconsistent: Chinese reviews include only Chinese-language trials and foreign reviews rarely incorporate Chinese studies, leading to partial viewpoints (14–17). A comprehensive NMA integrating multiple data sources has been lacking. Therefore, we pooled Chinese and English literature to compare ten clinically common sgAHs.

In this NMA, we combined direct and indirect evidence from 54 RCTs (17 English, 37 Chinese) of 7,290 participants with CU from both Chinese and English literature. Although the latest guidelines recommend the Urticaria Activity Score (UAS) to assess CU outcomes (1), most of the included trials still employed the TSS, which is calculated from pruritus severity and wheal counts (components also used in UAS) with minor scale variations. In theory, the two scores yield comparable clinical inferences; owing to scale heterogeneity, we synthesized effects with standardized mean differences (SMDs). Treatment duration was 2–4 weeks, in line with guideline advice. Risk of bias was evaluated with RoB 2.0: all trials claimed “randomization,” over half reported specific methods, but most ignored allocation concealment. Half of the studies were double-blind and patient adherence was generally good. Overall, six studies were judged high risk and 23 “some concerns,” with Chinese papers of lower quality than English ones. The analysis demonstrated that, with regard to the TSS, three sgAHs, olopatadine, mizolastine and bilastine were significantly more effective than placebo. For pruritus, all 10 agents outperformed placebo, statistical significance was reached by fexofenadine (SMD 2.56) and rupatadine (SMD 0.82). In probability rankings, fexofenadine, olopatadine, and rupatadine were ranked highest. These findings suggest that fexofenadine is the most effective agent for pruritus relief, with rupatadine representing a viable alternative. When wheal reduction was considered, ebastine proved superior to both mizolastine and loratadine, and apart from mizolastine, the remaining nine drugs all showed clear advantages over placebo. However, the finding that most agents failed to reach statistical significance and that mizolastine was ranked below placebo warrants further explanation.

An unexpected finding was that most second-generation antihistamines did not show statistically significant improvement in wheal control compared with placebo, and mizolastine ranked below placebo in the SUCRA analysis. This result should be interpreted with caution. Only 27 studies reported wheal scores, yielding a relatively sparse network and wider confidence intervals than those for TSS or pruritus. The limited statistical power may explain why several agents showed numerical but non-significant effects. In addition, significant inconsistency was detected in the wheal-score network (*p* < 0.001). This may reflect differences in baseline wheal severity, outcome assessment, or treatment duration across trials. The low ranking of mizolastine may therefore be related to limited direct evidence and trial-level heterogeneity, rather than definitive inferiority. These findings suggest that wheal-specific outcomes should be interpreted cautiously and should not be directly extrapolated from overall symptom improvement. Finally, the incidence of adverse events did not differ significantly between any active treatment and placebo, with fexofenadine displaying the lowest rate of side effects among the tested medications.

This NMA compared the efficacy and safety of 10 agents and provided a ranking to guide antihistamine selection. The inclusion of 37 Chinese-language and 17 English-language studies followed PRISMA recommendations with no language restrictions, rather than reflecting a deliberate focus on Chinese populations. The predominance of Chinese studies reflects the available evidence base for chronic urticaria, which is extensively studied in China. Excluding these studies would have introduced selection bias and reduced applicability to Asian populations. Although some Chinese trials had lower reporting quality for randomization and blinding, we addressed this through RoB 2.0 assessment and sensitivity analyses excluding high-risk studies, which supported the robustness of the main findings. Thus, including both Chinese and English studies strengthens the generalizability of our results.

Limitations include incomplete reporting of randomization and blinding in some trials; heterogeneous definitions of CSU, outcome measures and UAS reporting; omission of several commonly used drugs, such as astemizole and epinastine; and lack of evidence on up-dosed (up to four-fold) sgAHs, which current guidelines recommend when standard doses fail. We acknowledge the imbalance between Chinese- and English-language studies. However, this reflects the available evidence rather than selection bias, as no language restrictions were applied. Artificially balancing study numbers would itself introduce selection bias. To address quality concerns, we performed rigorous risk-of-bias assessment and sensitivity analyses.

In conclusion, olopatadine is most effective for reducing overall symptoms, fexofenadine best alleviates pruritus, ebastine excels at diminishing wheals, and fexofenadine has the lowest adverse-event rate. Nevertheless, beyond efficacy, clinicians should consider safety, cost, patient preference and local drug availability when selecting the optimal sgAHs in real-world practice.

## Data Availability

The original contributions presented in this study are included in the article/Supplementary material; further inquiries can be directed to the corresponding authors.
