# Supplementary material for: Second-generation H1 antihistamines for the treatment of chronic urticaria: a network meta-analysis
Source: Front Med (Lausanne). 2026 Aug 13;13:1880701. doi: 10.3389/fmed.2026.1880701 (PMC13520837; doi:10.3389/fmed.2026.1880701)
Supplement: Supplementary file 1 [file Table_1.doc]

| **Treatment comparison** | **Full model** |  |  | **Low-risk model** |  |  |
| --- | --- | --- | --- | --- | --- | --- |
|  | **Coefficient** | **SE** | **Pvalue** | **Coefficient** | **SE** | **Pvalue** |
| **B (Bilastine vs Placebo)** | **0.480** | **0.578** | **0.407** | **0.089** | **0.635** | **0.888** |
| **C (Cetirizine vs Placebo)** | **−0.004** | **0.767** | **0.996** | **−0.229** | **0.797** | **0.774** |
| **D (Desloratadine vs Placebo)** | **−0.312** | **0.699** | **0.655** | **−0.395** | **0.721** | **0.584** |
| **E (Ebastine vs Placebo)** | **−0.450** | **0.903** | **0.618** | **−0.682** | **0.937** | **0.467** |
| **F (Fexofenadine vs Placebo)** | **−0.263** | **0.543** | **0.629** | **−0.034** | **0.573** | **0.953** |
| **G (Levocetirizine vs Placebo)** | **0.312** | **0.667** | **0.640** | **0.252** | **0.692** | **0.716** |
| **H (Loratadine vs Placebo)** | **−0.536** | **0.706** | **0.448** | **−0.663** | **0.729** | **0.363** |
| **I (Mizolastine vs Placebo)** | **−1.190** | **0.805** | **0.140** | **−1.149** | **0.824** | **0.163** |
| **J (Placebo vs Placebo)** | **1.209** | **0.606** | **0.046** | **1.224** | **0.621** | **0.049** |
| **K (Rupatadine vs Placebo)** | **−0.247** | **0.694** | **0.722** | **−0.315** | **0.712** | **0.658** |
| **Tau²** | **0.928** | **0.180** | **<0.001** | **0.954** | **0.199** | **<0.001** |

**Table S1. Sensitivity analysis of the total symptom score (TSS) excluding studies with high risk of bias.**

| **Treatment comparison** | **Full model** |  |  | **Low-risk model** |  |  |
| --- | --- | --- | --- | --- | --- | --- |
|  | **Coefficient** | **SE** | **Pvalue** | **Coefficient** | **SE** | **Pvalue** |
| **B (Bilastine vs Placebo)** | **0.058** | **0.239** | **0.810** | **0.036** | **0.275** | **0.895** |
| **C (Cetirizine vs Placebo)** | **0.075** | **0.247** | **0.762** | **0.051** | **0.296** | **0.863** |
| **D (Desloratadine vs Placebo)** | **0.032** | **0.262** | **0.903** | **0.013** | **0.289** | **0.964** |
| **E (Ebastine vs Placebo)** | **0.270** | **0.305** | **0.376** | **0.291** | **0.328** | **0.375** |
| **F (Fexofenadine vs Placebo)** | **−0.052** | **0.200** | **0.796** | **−0.072** | **0.218** | **0.741** |
| **G (Levocetirizine vs Placebo)** | **0.126** | **0.279** | **0.653** | **0.111** | **0.304** | **0.714** |
| **H (Loratadine vs Placebo)** | **−0.027** | **0.267** | **0.920** | **−0.044** | **0.294** | **0.880** |
| **I (Mizolastine vs Placebo)** | **−0.885** | **0.441** | **0.045** | **−0.854** | **0.464** | **0.066** |
| **J (Placebo vs Placebo)** | **0.661** | **0.202** | **0.001** | **0.682** | **0.220** | **0.002** |
| **K (Rupatadine vs Placebo)** | **−0.255** | **0.251** | **0.309** | **−0.224** | **0.265** | **0.398** |
| **Tau²** | **0.311** | **0.067** | **<0.001** | **0.332** | **0.072** | **<0.001** |

**Table S2. Sensitivity analysis of the pruritus score excluding studies with high risk of bias.**

| **Treatment comparison** | **Full model** |  |  | **Low-risk model** |  |  |
| --- | --- | --- | --- | --- | --- | --- |
|  | **Coefficient** | **SE** | **Pvalue** | **Coefficient** | **SE** | **Pvalue** |
| **B (Bilastine vs Placebo)** | **0.165** | **0.544** | **0.761** | **0.192** | **0.560** | **0.732** |
| **C (Cetirizine vs Placebo)** | **0.183** | **0.659** | **0.781** | **0.213** | **0.662** | **0.747** |
| **D (Desloratadine vs Placebo)** | **−0.665** | **0.670** | **0.321** | **−0.597** | **0.672** | **0.375** |
| **E (Ebastine vs Placebo)** | **0.044** | **0.493** | **0.929** | **0.032** | **0.497** | **0.949** |
| **F (Fexofenadine vs Placebo)** | **0.288** | **0.687** | **0.675** | **0.411** | **0.691** | **0.552** |
| **G (Levocetirizine vs Placebo)** | **0.644** | **0.669** | **0.336** | **0.726** | **0.672** | **0.280** |
| **H (Loratadine vs Placebo)** | **−0.495** | **0.914** | **0.588** | **−0.251** | **0.924** | **0.786** |
| **I (Mizolastine vs Placebo)** | **0.542** | **0.571** | **0.343** | **0.538** | **0.572** | **0.347** |
| **J (Placebo vs Placebo)** | **−0.260** | **0.704** | **0.712** | **0.074** | **0.725** | **0.918** |
| **Tau²** | **0.839** | **0.149** | **<0.001** | **0.840** | **0.148** | **<0.001** |

**Table S3. Sensitivity analysis of wheal score excluding studies with high risk of bias.**

| **Treatment comparison** | **Full model** |  |  | **Low-risk model** |  |  |
| --- | --- | --- | --- | --- | --- | --- |
|  | **Log OR** | **SE** | **Pvalue** | **Log OR** | **SE** | **Pvalue** |
| **B (Bilastine vs Placebo)** | **0.543** | **0.436** | **0.214** | **0.383** | **0.449** | **0.393** |
| **C (Cetirizine vs Placebo)** | **0.519** | **0.426** | **0.224** | **0.436** | **0.443** | **0.325** |
| **D (Desloratadine vs Placebo)** | **0.095** | **0.455** | **0.834** | **0.051** | **0.460** | **0.911** |
| **E (Ebastine vs Placebo)** | **−0.313** | **0.426** | **0.463** | **−0.341** | **0.426** | **0.424** |
| **F (Fexofenadine vs Placebo)** | **0.279** | **0.355** | **0.433** | **0.263** | **0.360** | **0.465** |
| **G (Levocetirizine vs Placebo)** | **0.644** | **0.669** | **0.336** | **0.726** | **0.672** | **0.280** |
| **H (Loratadine vs Placebo)** | **0.327** | **0.426** | **0.442** | **0.281** | **0.430** | **0.514** |
| **I (Mizolastine vs Placebo)** | **0.001** | **0.466** | **0.999** | **−0.020** | **0.469** | **0.966** |
| **J (Placebo vs Placebo)** | **−0.011** | **0.329** | **0.972** | **−0.004** | **0.330** | **0.991** |
| **K (Rupatadine vs Placebo)** | **0.350** | **0.392** | **0.372** | **0.393** | **0.399** | **0.324** |
| **Tau²** | **0.391** | **0.106** | **0.0002** | **0.391** | **0.110** | **0.0004** |

**Table S4. Sensitivity analysis of the Incidence of adverse reactions excluding studies with high risk of bias.**
